# Lipocalin 2 promotes lung metastasis of murine breast cancer cells

**DOI:** 10.1186/1756-9966-27-83

**Published:** 2008-12-12

**Authors:** Han Shi, Yuchao Gu, Jing Yang, Liang Xu, Wenyi Mi, Wengong Yu

**Affiliations:** 1 Department of Molecular Biology, School of Medicine and Pharmacy, Ocean University of China, 5 Yushan Road, Qingdao, PR China

## Abstract

**Background:**

Lipocalin 2, an iron binding protein, is abnormally expressed in some malignant human cancers and may play an important role in tumor metastasis. However, the roles of lipocalin 2 in breast cancer formation and metastasis have not been clearly shown. This study aimed to investigate the roles of lipocalin 2 in breast tumor metastasis.

**Methods:**

Lipocalin 2 was overexpressed in the metastatic 4T1 murine mammary cancer cells. The effects of lipocalin 2 overexpression on the malignancy of breast cancer cells were examined using cell proliferation assay, migration assay, invasion assay, and soft agar assay *in vitro*. Tumor formation and metastasis abilities were examined using a well established mouse mammary tumor model *in vivo*.

**Results:**

Lipocalin 2 overexpression significantly enhanced the migration and invasion abilities of 4T1 cells *in vitro*, and lung metastasis *in vivo*. But overexpression of lipocalin 2 in 4T1 cells didn't affect cell proliferation and anchorage-independent growth *in vitro*, and primary tumor weight *in vivo*. Further studies demonstrated that the inhibition of the PI3K/Akt pathway could be a causative mechanism for the promotion of breast cancer migration/invasion induced by lipocalin 2 overexpression.

**Conclusion:**

These results clarified that lipocalin 2 could promote lung metastasis of 4T1 cells through the inhibition of the PI3K/Akt pathway, suggesting that lipocalin 2 was a potential target for therapy of breast cancer.

## Background

Lipocalins are a diverse family of over 20 small soluble, and often secreted, proteins. There is increasing evidence to claim that these proteins as transporters are involved in a variety of physiological functions. These functions include the regulation of immune responses, modulation of cell growth and metabolism, transportation of iron and prostaglandin synthesis [1]. Human NGAL (neutrophil gelatinase-associated lipocalin) and its mouse analogue lipocalin 2 (also referred to as siderocalin, Ngal, 24p3, uterocalin, or neu-related lipocalin) are members of the lipocalin family of small secreted proteins [2]. These proteins are up-regulated in a number of pathological conditions, including cancers, and may function as transporters of essential factors. Mouse lipocalin 2 was originally cloned from mouse kidney cells infected with polyoma virus-40 [3]. Yang *et al*. found lipocalin 2 could bind to iron and then deliver it to the cell through a process requiring endocytosis [4]. Subsequently, lipocalin 2 cell-surface receptor 24p3R was identified [5]. Moreover, lipocalin 2 was induced by lipopolysaccharide [6], basic fibroblast growth factor [7], tumor necrosis factor α [8] and retinoic acid [9]. Until now, growing evidence suggests that lipocalin 2 plays an important role in innate immune response, cell apoptosis and tumor development [10].

In human, elevated levels of lipocalin 2 expression have been reported in various cancers including ovarian cancer, pancreatic cancer, lung cancer, colon cancer and breast cancer, indicating there is a strong association between lipocalin 2 and the malignance of cancer cells and that metastasis [11]. It was found that NGAL/lipocalin 2 was overexpressed in the progression of malignant transformation from human immortalized esophageal epithelial cell line SHEE to esophageal carcinoma cell line SHEEC [12]. Moderate to strong expression of NGAL/lipocalin 2 was observed in epithelial ovarian cancer cell lines SKOV3 and OVCA433 while no expression of NGAL/lipocalin 2 was evident in normal IOSE29 and mesenchyme-like OVHS1, PEO.36 and HEY cell lines [13]. A study of Fernandez *et al*. demonstrated that in MCF-7 human breast cancer cells lipocalin 2 could enhance tumor growth and metastasis by protecting matrix metalloproteinase-9 (MMP-9) from degradation and increasing angiogenesis. And, the MMP-9/lipocalin 2 complexes were detected in 90% of urine samples obtained from breast cancer patients, but not in those from healthy controls [14]. Besides that, cell lines derived from highly metastatic breast cancer, such as MDA-MB-231, expressed and secreted higher amounts of NGAL/lipocalin 2 than cell lines derived from benign, organ defined breast cancers [15].

Mouse lipocalin 2 is overexpressed in oncogene-mediated cell transformation [16]. Under normal conditions, expression of lipocalin 2 is restricted to breast [17]; however, increased lipocalin 2 levels have been reported in breast cancer [18]. Although lipocalin 2 have been demonstrated to correlate with breast cancer [19], the roles of lipocalin 2 in breast cancer formation and metastasis have not been clearly shown. In this study, we overexpressed lipocalin 2 in 4T1 mouse mammary tumor cells, and investigated the effects and molecular mechanisms of lipocalin 2 on breast tumor malignant properties.

## Methods

### Cells and cell culture

4T1 is mouse mammary tumor cell line [20], which was kindly provided by Dr. Fred R. Miller at the Karmanos Cancer Institute in Detroit, MI. 4T1 is cultured in Dulbecco's modified Eagle's medium (DMEM) supplemented with 10% fetal calf serum (DME-10), 1 mM mixed nonessential amino acids, and 2 mM L-glutamine.

### Plasmid construction and establishment of stable transfectants

To create the mouse lipocalin 2 overexpression vector, full-length lipocalin 2 encoding gene was amplified from the total RNA of 4T1 cells with RT-PCR (the cloning primer: lipocalin2F: 5'-GAAGATCTATGGCCCTGAGTGTCATGTG-3', lipocalin2R: 5'-GGAATTCTCAGTTGTCAATGCATTGG-3'). The gene was then digested by *Bgl*II/*EcoR*I and inserted into *Bgl*II/*EcoR*I double digested pMSCVpuro (a self-inactivating murine stem cell virus plasmid) vector, resulting in the lipocalin 2 retroviral expression vector, pMSCVpuro-lipocalin2.

Retrovirus was produced in the Phoenix packaging cell line. In brief, Phoenix cells were plated at 2 × 10^6 ^cells/well in 60-mm plates and allowed to adhere overnight. The cells were separately transfected with the pMSCVpuro or pMSCVpuro-lipocalin2 plasmid (10 μg/plate) by CaCl_2 _transfection. Replication retrovirus was harvested 48 hours after transfection, sterile filtered to remove nonadherent producer cells, and then infected 4T1 cells separately. Infected cells were cultured in medium with 4 μg/ml puromycin for 2 weeks. The resistant clones were isolated by limit dilution and dispatched in new dishes. Then, the obtained cells were named Mock and lipocalin 2 (LCN2) respectively.

### RNA isolation and semiquantitative RT-PCR (Reverse transcription polymerase chain reaction)

Total RNA was isolated from cells using Trizol (Invitrogen, Carlsbad, CA, USA) and reverse transcription was carried out using High Capacity cDNA Archive Kit (Applied Biosystems, Foster City, CA, USA). The relative quantitative analysis was normalized to endogenous control β-actin. Mouse lipocalin 2 forward primer was 5'-TGCAGGTGGTACGTTGTGG-3', and its reverse primer was 5'-TGTTGTCGTCCTTGAGGC-3'. Mouse β-actin forward primer was 5'-ATCTGGCACCACACCTTCTAC-3', and its reverse primer was 5'-CACACTTCATGATGGAATTGAA-3'.

### Cell proliferation assay

Cells were seeded 1 × 10^4 ^per well in a 96-well plate. Cells were allowed to grow for 24 hours. Then, 20 μl of 3-(4,5-dimethylthiazol-2-yl)-2,5-diphenyltetrazolium bromide (MTT) (5 mg/ml) was added to each well. After 4 hours of incubation at 37°C, cells were lysed by addition of 200 μl dimethylsulfoxide (DMSO). Absorbance was measured at 570 nm using a Rainbow microplate reader (Tecan, Groding/Salzburg, Austria).

### Assays for soft agar colony

Colony formation in soft agar was assessed as described [21]. Cells (5 × 10^3^) from Mock and LCN2 were suspended in 1 ml top agar medium (DME-10 supplied with 0.4% agar), in the presence or absence of phosphoinositide-3 kinase (PI3K)-specific inhibitor LY294002 (Merck, Nottingham, UK), and layered over 1.5 ml bottom agar medium (DME-10 supplied with 0.8% agar) in 35-mm dishes. After 3 weeks, the cells were photographed under inverted microscope and the number of colonies was counted. Independent experiments were performed in triplicates.

### *In vitro* migration assay and invasion assay

Cell migration was assayed using Transwell chambers (6.5 mm; Corning, New York, USA) with 8 μm pore membranes. The lower chamber was filled with 600 μl NIH-3T3 conditioned medium [22] containing 20 μg/ml fibronectin (BD Biosciences, Bedford, MA, USA) with or without 2 μM LY294002. Cells (5 × 10^4^) were suspended with 100 μl upper medium (DMEM with 1% fetal calf serum) and planted into the upper chamber with or without 2 μM LY294002. After 16 hours, the number of cells appearing by crystal violet staining on the undersurface of the polycarbonate membranes was scored visually in five random fields at 100× magnification using a light microscope.

For invasion assay, the upper face of the membrane was covered with 70 μl Matrigel (1 mg/ml; BD Biosciences). The invasion assay procedure was the same as for the migration assay, except that the incubation time of the experiment was prolonged to 24 hours.

### Primary tumor growth and lung metastases assay

These procedures were performed as described previously [20,23] with minor modifications. Female BALB/c mice, aged 8 to 10 weeks, were used in the experiment. In brief, mice (six to eight per group) were anesthetized with sodium pentobarbital (50 mg/kg body weight), and tumor cells (5 × 10^5^) in 10 μl DME-10 were injected into the mammary gland. The weight of the primary tumors and the number of metastatic nodules on the lung surface were evaluated 30 days after the tumor cells injection. The animals were housed and cared for in accordance with the guidelines established by the National Science Council of Republic of China.

### Immunoprecipitation (IP) and Immunoblotting (IB) analysis

Cells were lysed in lysis buffer (50 mM Tris-HCl [pH 7.4], 150 mM NaCl, 1% NP40, 1 mM EDTA, 1 mM Na_3_VO_4_, 10 mM NaF) containing a protease inhibitor cocktail (Roche, Nutley, NJ, USA). Protein samples (50 μg) were separated by 12% SDS-PAGE and transferred to Immobilon-P membranes (Millipore, Bedford, MA, USA). Antibodies to phosphorylated and total Akt (Cell Signaling, Beverly, MA, USA), phosphorylated (Ser380/Thr382/383) and total PTEN (Cell Signaling), phosphorylated (Thr202/Tyr204) and total MAPK (Cell Signaling), and β-Actin (Santa Cruz, CA, USA) were used, with detection by ECL-detecting reagent (Amersham Biosciences, Buckinghamshire, UK). Quantification of the blots was conducted using Image-Pro Plus software (version 6.0; Media Cybernetics, Bethesda, MD, USA).

For immunoprecipitation assay, after cells were cultured for 48 hour, we collected 1 ml of the DMEM medium for immunoprecipitation with anti-mouse lipocalin 2 monoclonal antibody (Santa Cruz). The following procedure was the same as IB.

### Statistic analysis

All of the results were repeated in at least three independent experiments and consistently yielded similar results. Data was presented as mean ± SD. Statistical significance was analyzed using the SPSS 11.0 software program (SPSS Inc., Chicago, IL, USA). The value of *P *< 0.05 was considered statistically significant.

## Results

### Overexpression of lipocalin 2 gene in 4T1 cell line

**Figure 1:**
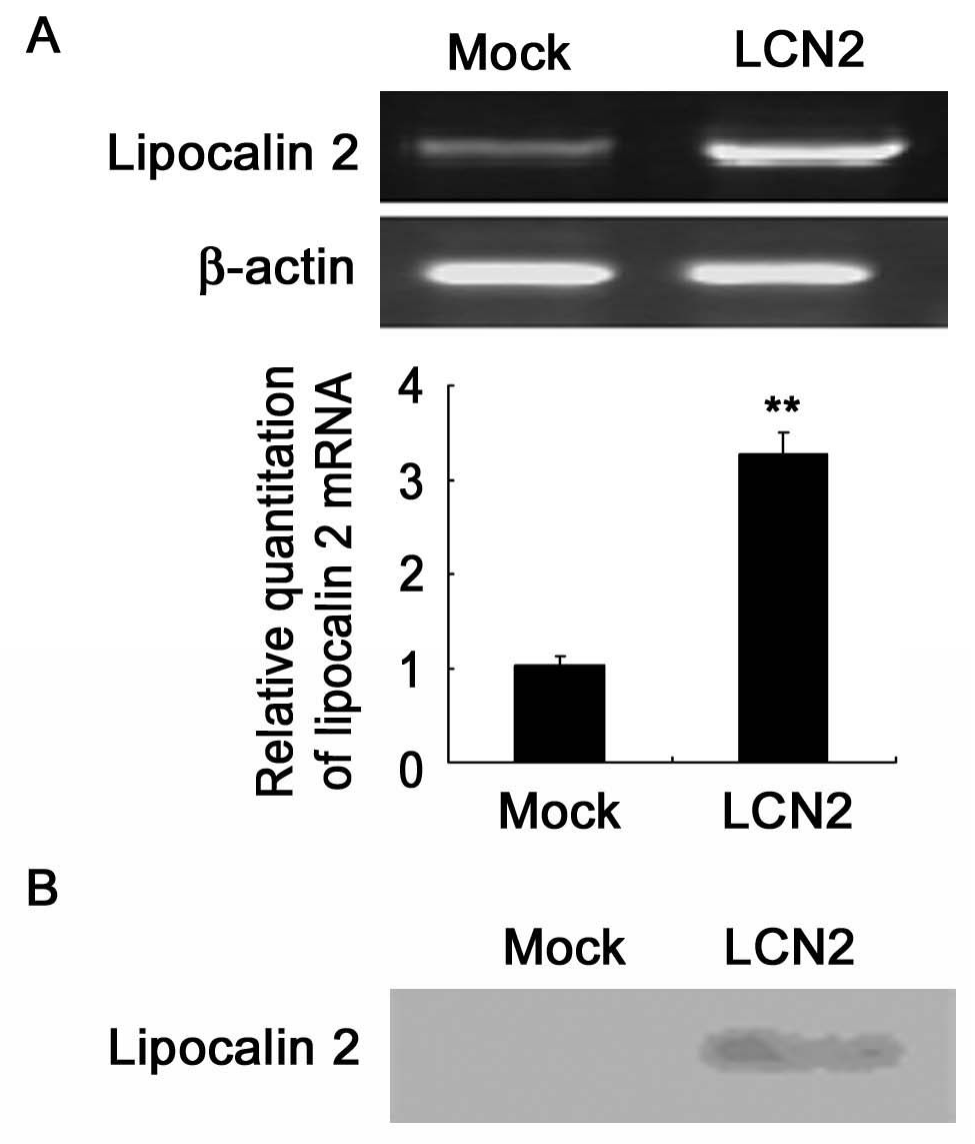
**Overexpression of lipocalin 2 in 4T1 cells**. (A) Upper panels: semiquantitative RT-PCR analysis of lipocalin 2 mRNA expression in LCN2 and Mock cells. Lower panels: quantitation of lipocalin 2 after normalization with the β-actin. (B) Western blotting analysis was performed with antibody recognizing mouse lipocalin 2. **P < 0.01.

To determine whether lipocalin 2 played an important role in tumor metastasis, lipocalin 2 gene was stably overexpressed in 4T1 cell line. As shown in Figure 1A, the expression of lipocalin 2 mRNA was increased obviously in 4T1 cells infected with pMSCVpuro-lipocalin2 (LCN2), compared to that in 4T1 cells infected with pMSCVpuro (Mock); and the secreted lipocalin 2 level was increased significantly in the conditional medium of LCN2 (Figure 1B).

### Effects of lipocalin 2 on proliferation and anchorage-independent growth of 4T1 cells

**Figure 2:**
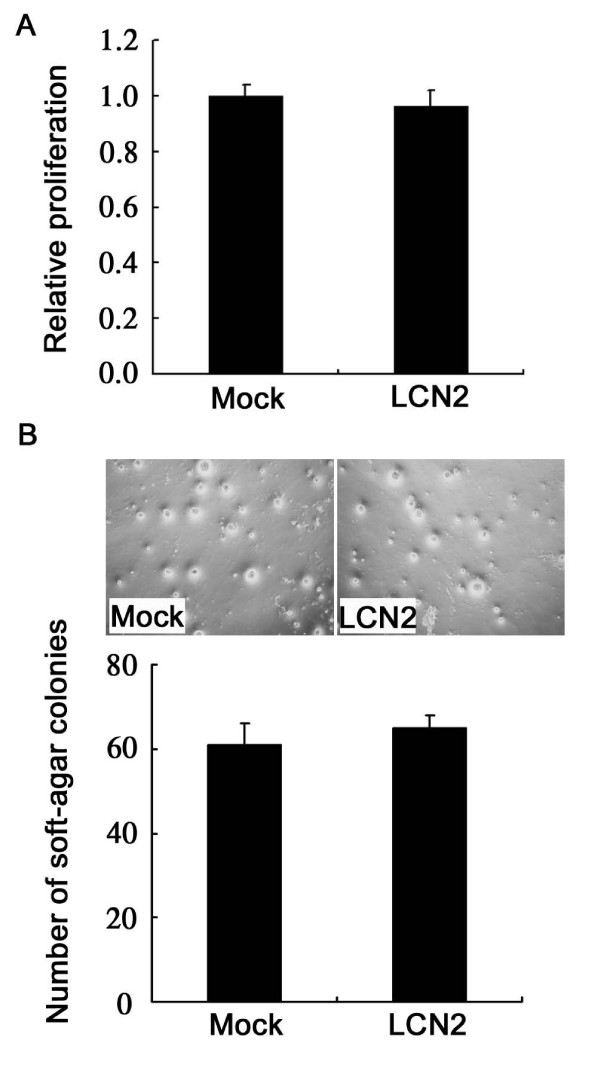
**Analysis of proliferation and anchorage-independent growth of 4T1 cells**. (A) The proliferation of lipocalin 2 overexpressed 4T1 cells was analyzed by MTT assay. (B) Anchorage-independent growth of lipocalin 2 overexpressed 4T1 cells was analyzed by soft agar assay.

To investigate whether lipocalin 2 overexpression in 4T1 cells affected their proliferation, MTT assay was performed. As shown in Figure 2A, lipocalin 2 did not affect the proliferation of 4T1 cells. As anchorage-independent growth measured by colony formation in a semi-solid medium was thought to be one of the fundamental properties of malignant cells, we further investigated the ability of anchorage-independent growth. The result indicated that LCN2 cells and Mock cells formed the same colonies (Figure 2B).

### Lipocalin 2 enhances migration and invasion of 4T1 cells *in vitro*

**Figure 3:**
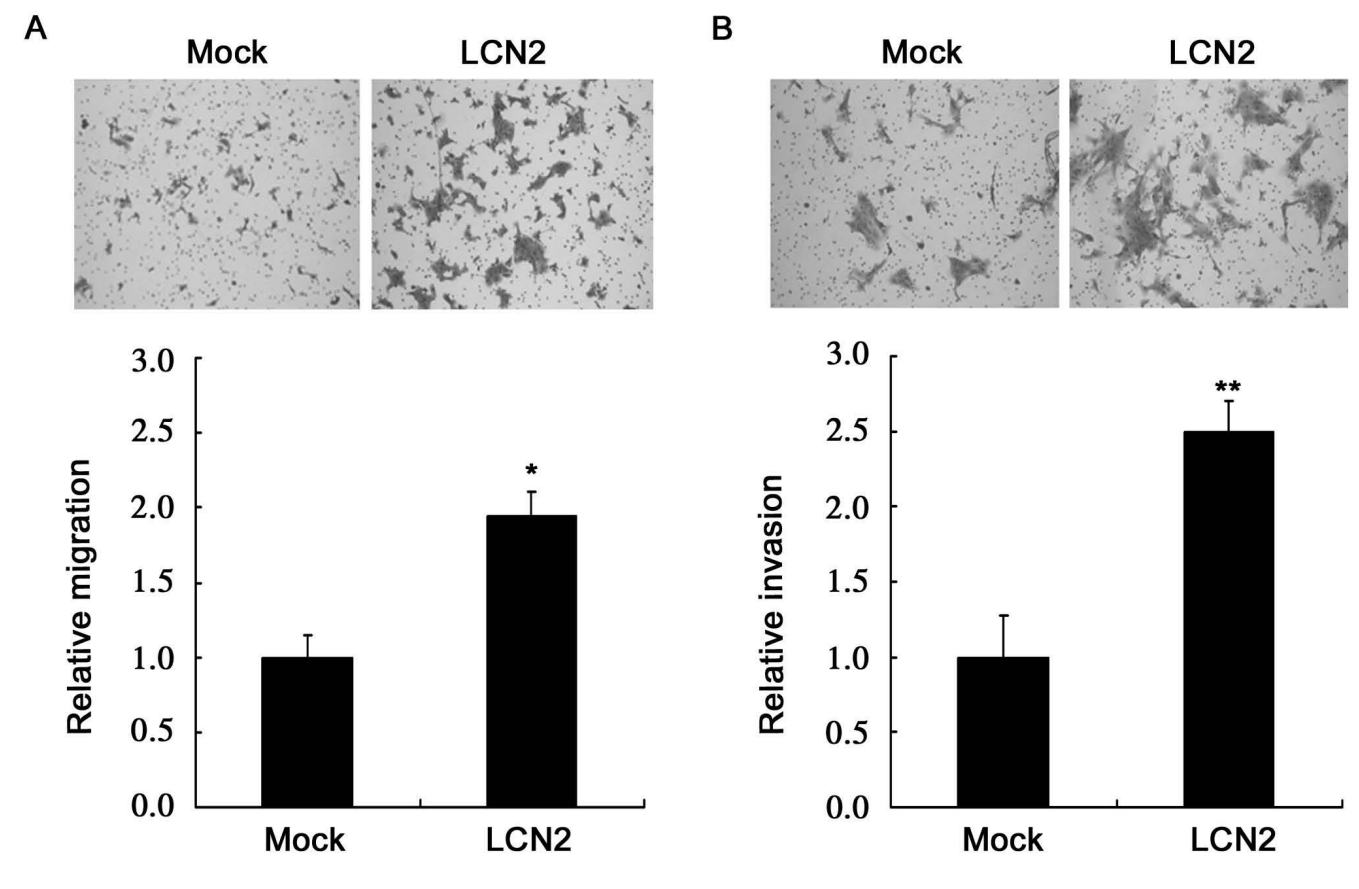
**Effects of lipocalin 2 on migration and invasion of 4T1 cells**. (A) Transmembrane cell migration. Motility of Mock and LCN2 cells was evaluated by Transwell migration assays. Relative cell migration was determined by the number of the migrated cells normalized to the total number of the cells adhering to well and the value from Mock cells was arbitrarily set at 1. (B) The relative invasion assays for lipocalin 2 overexpressed 4T1 cells. *P < 0.05, **P < 0.01.

To investigate the role of lipocalin 2 in 4T1 cells metastasis, we determined the effects of lipocalin 2 on the migration and invasion of 4T1 cells *in vitro*. As shown in Figure 3, the overexpression of lipocalin 2 significantly promoted 4T1 cells migration and invasion.

### Lipocalin 2 promotes lung metastases of 4T1 cells *in vivo*

**Figure 4:**
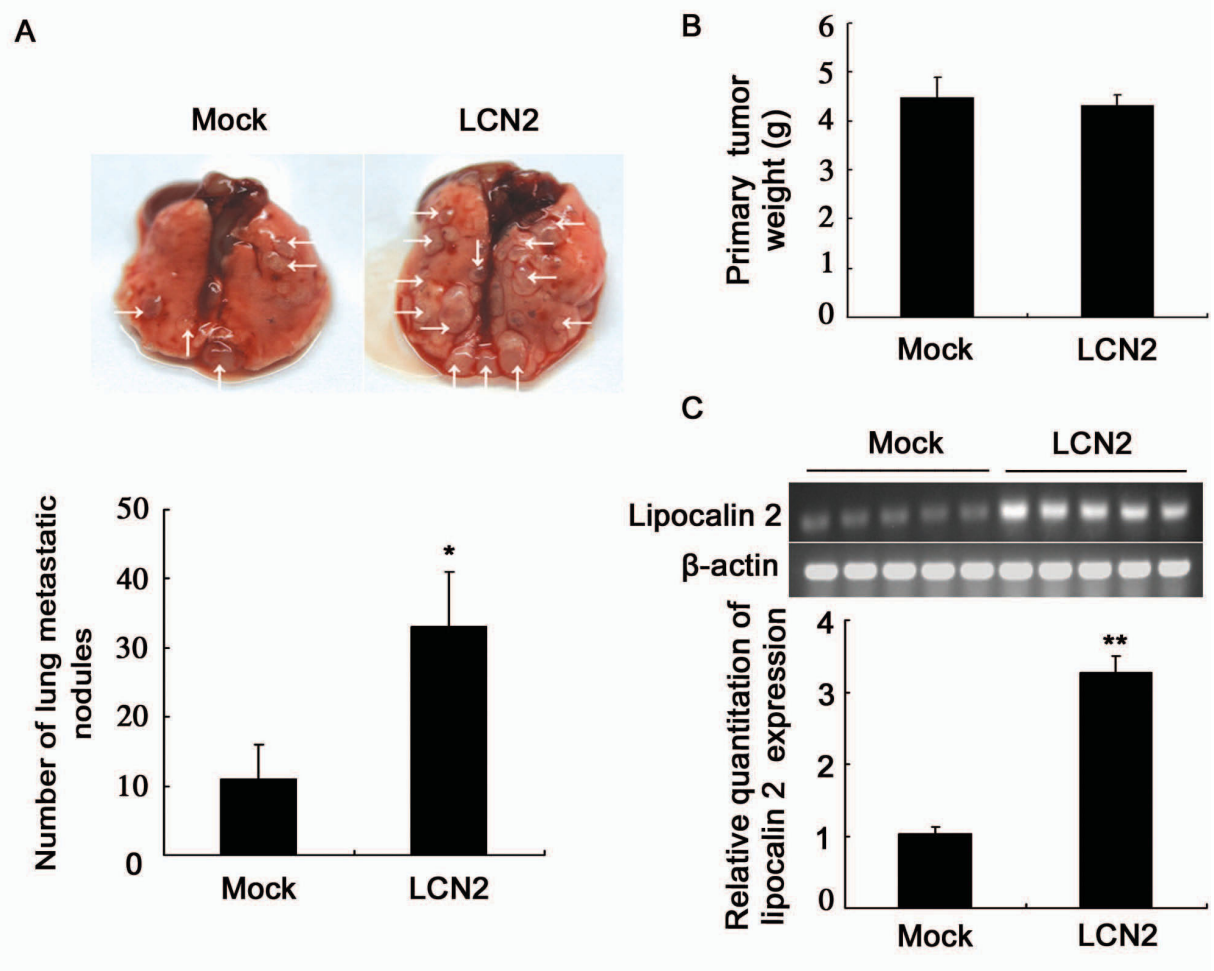
**Effects of lipocalin 2 on tumor formation and lung metastases**. Murine were killed and analyzed at the end of 30 days post tumor implantation. (A) Upper panels: representative photos of the lungs. The arrows point to the metastatic nodules in lungs. Lower panels: the average numbers of lung metastatic nodules. (B) Primary tumor weights. (C) Upper panels: semiquantitative RT-PCR analysis of lipocalin 2 expression in primary tumors. Lower panels: quantitation of lipocalin 2 was performed after normalization with the β-actin. *P < 0.05, **P < 0.01.

Based on the roles of lipocalin 2 in the migration and invasion of 4T1 cells as described above, we next examined the effects of lipocalin 2 on tumor formation and metastasis *in vivo*. The results demonstrated that lipocalin 2 dramatically increased the number of visible metastatic nodules on the lung surface of tumor-bearing mice (Figure 4A), but had no effect on the primary tumor weight (Figure 4B). Then the expression level of lipocalin 2 in primary tumors was investigated. Consistent with the results in Figure 1A, Figure 4C showed that the expression of lipocalin 2 in LCN2 tumors was higher than that in Mock tumors. These results indicated that lipocalin 2 remained stable in both Mock and LCN2 tumors, and had no effect on tumor growth.

### Lipocalin 2 promotes breast cancer cells migration and invasion through PI3K/Akt pathway

**Figure 5:**
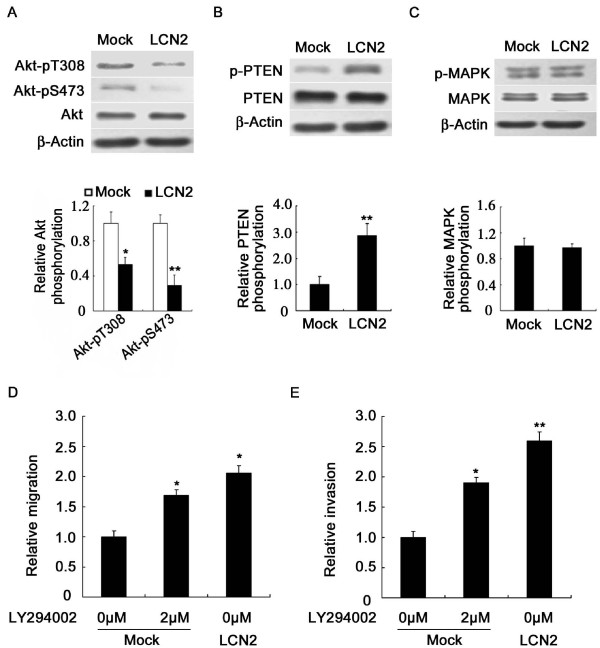
**Lipocalin 2 promotes breast cancer cell migration through suppressing PI3K/Akt pathway**. (A) Upper panels: the expression and activation of Akt detected by immunoblotting with anti-Akt, anti-p-Akt Ser (473), and anti-p-Akt Thr (308) antibodies. Lower panels: quantitation of these blots after normalization with the β-Actin blot. (B) Upper panels: the expression and activation of PTEN detected by immunoblotting. Lower panels: quantitation of these blots after normalization with the β-Actin blot. (C) Upper panels: expression and activation of MAPK detected by immunoblotting. Lower panels: quantitation of these blots after normalization with the β-Actin blot. (D) The migration ability of 4T1 cells was enhanced by LY294002. (E) The invasion ability of 4T1 cells was enhanced by LY294002. *P < 0.05, **P < 0.01. p-MAPK, phosphor-MAPK Thr202/Tyr204; MAPK, mitogen-activated protein kinase; p-PTEN, phosphor-PTEN Ser380/Thr382/383; PTEN, phosphatase and tensin homolog; PI3K, phosphoinositide-3 kinase.

To investigate the molecular mechanism of lipocalin 2-mediated cells migration and invasion, some tumor metastasis-associated proteins and signaling pathways were assayed by IB. As shown in Figure 5, the overexpression of lipocalin 2 significantly decreased the phosphorylation of Akt at two key residues Thr308 and Ser473 (Figure 5A), and increased the phosphorylation of PTEN in 4T1 cells (Figure 5B); however, the overexpression of lipocalin 2 did not affect the expression and phosphorylation of MAPK (Figure 5C).

Finally, we evaluated whether signaling through the PI3K/Akt pathway impacted cell migration/invasion. The results showed that PI3K specific inhibitor LY294002 (2 μM) accelerated migration/invasion of Mock cells, similar to the effects induced by lipocalin 2 overexpression (Figure 5D and 5E). These results suggested that lipocalin 2 overexpression could cause an increase of PTEN activation and result in the inhibition of Akt signaling, which promoted cell migration and invasion.

### PI3K/Akt pathway has no effect on anchorage-independent growth of breast tumor cells

**Figure 6:**
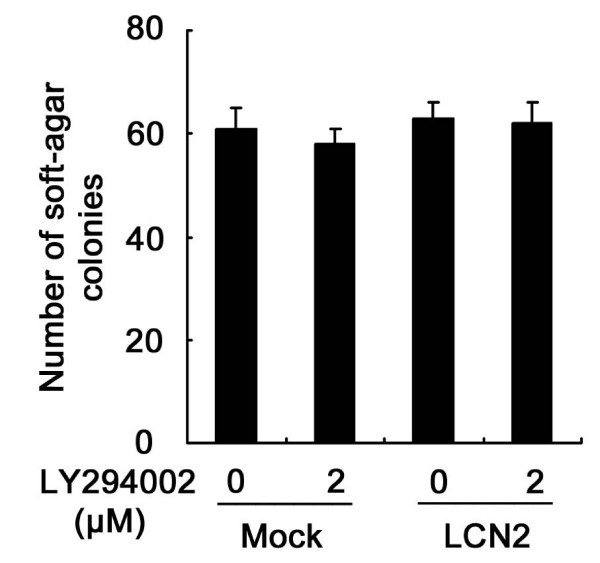
**Effect of PI3K/Akt signal on the anchorage-independent growth of breast cancer cells**. The anchorage-independent growth of both LCN2 and Mock (5 × 10^3 ^cells/well) were not affected by the inhibition of the PI3K/Akt pathway. PI3K, phosphoinositide-3 kinase.

To determine whether activation of the PI3K/Akt pathway was responsible for the anchorage-independent growth affected by lipocalin 2 overexpression in 4T1 cells, we inactivated the PI3K/Akt pathway with LY294002. The colony forming abilities of both lipocalin 2-overexpression 4T1 cells and the Mock cells were unaffected by inhibition of the PI3K/Akt pathway (Figure 6), indicating that the anchorage-independent growth caused by lipocalin 2 was not associated with the PI3K/Akt pathway.

## Discussion

Breast cancer is a major problem for public health. In women with breast cancer, it is not the primary tumor but its metastasis to distant sites that is the ultimate cause of death. Therefore, the identification of new markers as well as the definition of new therapeutic targets is of critical importance [24].

In our paper, the findings demonstrated that lipocalin 2 didn't affect the proliferation and anchorage-independent growth of 4T1 cells *in vitro *and primary tumor weight *in vivo*. Some studies about the roles of lipocalin 2 in cancer progression supported our findings. For example, lipocalin 2 expression level was found to be significantly higher in oesophageal squamous cell carcinoma (ESCC) than in normal mucosa. However, no significant association was observed between lipocalin 2 expression and cell proliferation [25]. Moreover, another report also indicated that lipocalin 2 was possibly involved in invasion of tumor cells by regulating activity of MMP-9 and MMP-2, but was not apparently related with division and proliferation of tumor cells in SHEEC [12]. These studies suggested that lipocalin 2 expression wasn't related with cancer proliferation.

The process of tumor metastasis is a multistage event involving local invasion and destruction of intercellular matrix, intravasation into blood vessels, lymphatics or other channels of transport, survival in the circulation, extravasation out of the vessels in the secondary site and growth in the new location [26]. In our study, although the effects of lipocalin 2 on proliferation and anchorage-independent growth of 4T1 cells were not significant, our results from cell migration and invasion analysis indicated that cell mobility was significantly higher in the LCN2 cells than Mock cells, denoting an aggressive phenotype in tumor cells. Because increased malignant cell motility has been associated with enhanced metastatic potential in animal as well as human tumors [27], we further did an experiment *in vivo*. As we predicted, the results revealed that lipocalin 2 overexpression enhanced the metastasis of 4T1 cells in BALB/c mice. This enhancement suggested that lipocalin 2 might provide some advantage to the cancer cells growing in the complex cellular environment of the host tissues. Although the precise nature of this advantage remains to be determined, previous studies have argued to suggest that lipocalin 2 may modulate the interactions with immune system. For example, it has been demonstrated that lipocalin 2 synthesis is highly induced in epithelial cells in both inflammatory and neoplastic colorectal diseases [28]. Lipocalin 2 may also bind other lipophilic mediators of inflammatory responses and extracellular matrix-degrading proteinases, which jointly cause invasion and aggravation. Our results suggested that lipocalin 2 was a key modulator for breast cancer cells metastasis.

The previous report indicated that lipocalin 2 could diminish migration and invasion of 4T1-Ras-transformed mesenchymal tumor cell line [29]. And a study by Venkatesha *et al*. demonstrated that lipocalin 2 could antagonize the proangiogenic action of Ras-transformed cells [2]. These results indicated that lipocalin 2 was an epithelial inducer in Ras malignancy and a suppressor of metastasis. In contrast, our results showed that lipocalin 2 promoted lung metastasis in non-Ras-induced 4T1 cells. Therefore, the effect of lipocalin 2 on tumor metastasis depended on the tumor-type specificity. It was necessary to define the roles and molecular mechanisms of lipocalin 2 in given cancer metastasis. To gain a better understanding of these metastasis effects, the molecular mechanisms by which lipocalin 2 affected cell migration and invasion should be studied. The previous study reported that the Ras-MAPK and the PI3K/Akt pathways were critical for the maintenance of EMT (epithelial to mesenchymal transition) in 4T1-Ras cells [29]. It also demonstrated that lipocalin 2 inhibited Ras-mediated invasion/migration by up-regulating E-cadherin through an inhibition of MAPK signaling. But our study found that overexpression of lipocalin 2 could not affect the MAPK pathway. It is generally accepted that the PI3K/Akt axis promotes tumorigenesis by increasing the survival capacity of cancer cells [30]. However, Gu *et al*. demonstrated the PI3K/Akt pathway had no effect on anchorage-independent growth of 4T1 cells [31]. Here, we also found the colony forming abilities of both Mock cells and LCN2 cells were unaffected by inhibition of the PI3K/Akt pathway, indicating this reduction of Akt activity caused by lipocalin 2 had no effect on survival of 4T1 cells. Besides that, some recently published evidence indicated that Akt could block breast cancer cell migration and invasion [31-34]. In this study, we found that the overexpression of lipocalin 2 increased migration and invasion of non-Ras-induced 4T1 cells and inhibited the PI3K/Akt pathway in non-Ras-induced 4T1 cells. Altogether, lipocalin 2 is associated with breast cancer cells migration and invasion occurred, at least partly, through the PI3K/Akt pathway, although further studies will be necessary to confirm this finding.

## Conclusion

This study demonstrated that lipocalin 2 overexpression could increase cell migration, invasion, and lung metastasis in 4T1 murine breast cancer cells. The molecular mechanism underlying the lipocalin 2-mediated migration and invasion was found to be inhibition of the PI3K/Akt pathway. To our knowledge, this report is the first to elucidate the involvement of lipocalin 2 and mechanisms by which it influences the malignant properties of breast cancer cells.

## Competing interests

The authors declare that they have no competing interests.

## Authors' contributions

HS participated in designing the study, conducted cell line transfection, immunoblotting analysis, cell and animal experiments, and drafted the manuscript. YG participated in the design of the study and conducted cell line transfection. JY participated in vector construction. LX and WM conducted the immunoblotting analysis. WY conceived of the study, participated in its design and coordination, and helped to draft the manuscript. All authors read and approved the final manuscript.
